# Cancer cells and viruses share common glycoepitopes: exciting opportunities toward combined treatments

**DOI:** 10.3389/fimmu.2024.1292588

**Published:** 2024-03-01

**Authors:** René Roy

**Affiliations:** Glycosciences and Nanomaterial Laboratory, Université du Québec à Montréal, Montréal, QC, Canada

**Keywords:** O-glycans, vaccines, cancer, viruses, glycobiology, glycoepitopes, tumor associated carbohydrate antigens (TACAs), SARS-CoV-2

## Abstract

Aberrant glycosylation patterns of glycoproteins and glycolipids have long been recognized as one the major hallmarks of cancer cells that has led to numerous glycoconjugate vaccine attempts. These abnormal glycosylation profiles mostly originate from the lack of key glycosyltransferases activities, mutations, over expressions, or modifications of the requisite chaperone for functional folding. Due to their relative structural simplicity, *O*-linked glycans of the altered mucin family of glycoproteins have been particularly attractive in the design of tumor associated carbohydrate-based vaccines. Several such glycoconjugate vaccine formulations have generated potent monoclonal anti-carbohydrate antibodies useful as diagnostic and immunotherapies in the fight against cancer. Paradoxically, glycoproteins related to enveloped viruses also express analogous *N*- and *O*-linked glycosylation patterns. However, due to the fact that viruses are not equipped with the appropriate glycosyl enzyme machinery, they need to hijack that of the infected host cells. Although the resulting *N*-linked glycans are very similar to those of normal cells, some of their *O*-linked glycan patterns often share the common structural simplicity to those identified on tumor cells. Consequently, given that both cancer cells and viral glycoproteins share both common *N*- and *O*-linked glycoepitopes, glycoconjugate vaccines could be highly attractive to generate potent immune responses to target both conditions.

## 1 Introduction

Tumor-associated carbohydrate antigens (TACAs) have long been recognized as key targets toward prophylactic vaccines against cancer (1–5). The corresponding glycoepitopes originate from either glycolipids and gangliosides in particular (6–8) or from their glycoprotein counterparts, in particular cell-surface mucins (9). They are all constitutive members of the glycan structures found on the extracellular membranes of cells where they orchestrate cellular recognition, adhesion, and signaling (10) as well as several other functions, not yet fully exploited (11). As such, carbohydrates add to the arsenal of encoded information analogous to those attributed to nucleotides and amino acids, now referred to as glycocodes (12). Therefore, and not surprisingly, the overall information encoded by carbohydrates holds great promises for cancer (13) and cancer-associated glycosylation for the discovery of effective new cancer drugs (14, 15). As a result, TACAs are important members of the prioritization of cancer antigens which have been included in the National Cancer Institute (NCI) pilot project for accelerated translational research (16). Unfortunately, the targeting of *O*-linked tumor glycans for generating anti-cancer vaccines has had limited successes in clinical Phase III trials (17) and a thorough analysis of the *post mortem* data might be useful to explain the numerous failures (18).

## 2 Glycan biosynthesis and structures of TACAs on tumor cells

**Figure 1:**
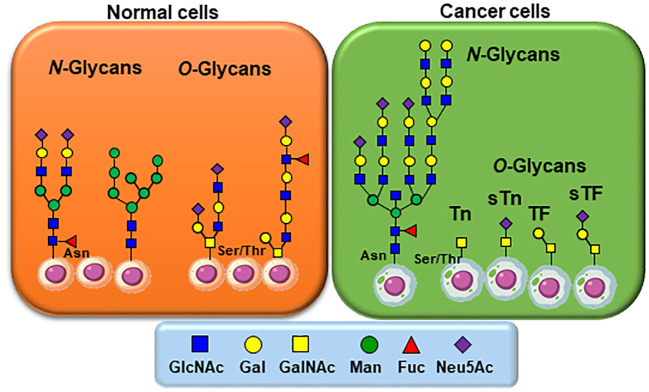
Typical glycan structures as they usually appear on normal and cancer cells. For *N*-linked glycans, altered and increased branching and/or capping by fucosylation and sialylation occurs. Of particular interest is the most simplified structural architectures of *O*-linked glycans in comparison to those identified on healthy cells. *N*-linked carbohydrates on glycoproteins are built from β-*N*-acetylglucosamine (GlcNAc) attached to asparagine (Asn) while *O*-linked glycoproteins are initiated by an α-*N*-acetylgalactosamine (GalNAc) to either Serine or Threonine (Ser/Thr).

As mentioned, the transformation from normal to malignant phenotype in human cancers is associated with aberrant cell surface glycosylation (**Figure 1**) (13–15). The biosynthesis of *O*-linked glycans is initiated and completed in the Golgi apparatus. In normal cells, mucin-type *O*-glycans are represented by structurally complex branched and linear arrangements of monosaccharides that are sequentially assembled by appropriate glycosyltransferases to glycoproteins on serine/threonine residues within the Golgi apparatus. The synthesis of mucin-type *O*-glycans is complex and depends on many factors. Alternatively, the mucin-type *O*-glycans are constituted by shorter saccharide sequences in cancer cells due to glycoenzymes mis-regulations.

*N*-linked carbohydrates on glycoproteins are built in three key steps from a lipid-linked (dolichol-phosphate) α-*N*-acetylglucosamine (GlcNAc) residue to which multiple saccharide chains are attached for transfer, followed by a transfer to nascent proteins through attachment to asparagine residues via an inverted β-GlcNAc linkage, and final processing of complex glycan chains (19, 20). Sialylated Lewis X, over sialylated glycans (21), together with the most common polylactosamine elongation, represent typical members of this class of TACAs (22).

**Figure 2:**
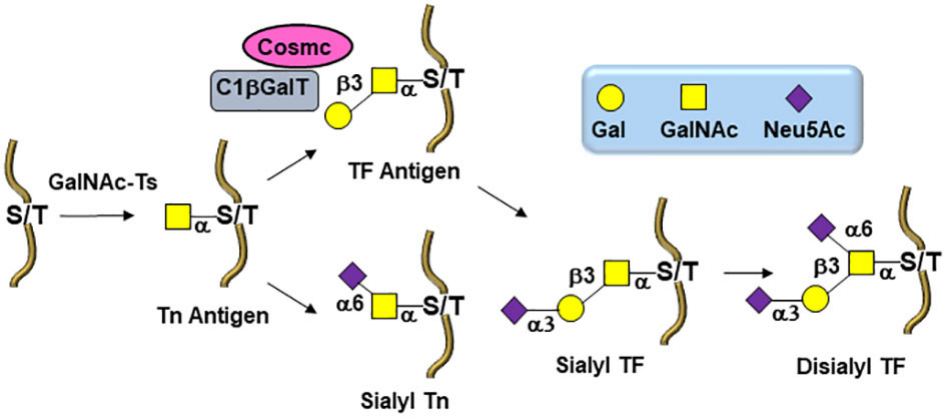
Biosynthetic pathways leading to *O*-linked tumor-associated carbohydrate antigens (TACAs) in tumor cells.

The biosynthesis of *O*-linked glycans, pertinent to this report and their relation to viruses, are rather initiated by an α-*N*-acetylgalactosamine (GalNAc) linked to either Serine or Threonine (GalNAc-Ser/Thr) that is also a post-translational modification that occurs after the protein has been synthesized. Overall and in both cases, *N*-/*O*-glycosylation is the most commonly occurring post-translational protein modifications. A malfunctioning in glycan processing is due to the lack of key glycosyltransferases activities, mutations, over expressions, or modifications of the requisite chaperone for functional folding. Alteration of several types of *O*-glycan core structures has been widely implicated in multiple forms of cancers (**Figure 2**) (23–28). Approximately twenty GalNAc-transferase isoenzymes (GalNAc-Ts) have been identified in this initial key step (29). They are organized into nine subfamilies according to the similarity of their sequences. GalNAc-Ts select their sites of *O*-linked glycosylation depending on several structural differentiations of the peptide sequences. The biosynthesis of the ensuing abnormally *O*-glycosylated proteins is schematically illustrated in **Figure 2**. Elongation to core-2 glycans is prohibited by loss of an important β-GlcNAc transferase activity, resulting in buildup of the core-1 disaccharide, also known as the Thomsen Friedenreich (TF) antigen, Galβ1-3GalNAcα1-Ser/Thr. Biosynthesis of the TF antigen is through an essential C1βGal transferase, whose activity is dependent on the molecular chaperone called *Cosmc.* Mutation of *Cosmc* halts mucin type glycosylation at a single α-GalNAc residue, also known as the Thomsen nouveau or Tn antigen. Consequently, the buildup of the so-called *O*-linked monosaccharide Tn antigen (GalNAcα1-Ser/Thr) is overexpressed. The over expression of the ensuing Thomsen–Friedenreich disaccharide antigen (Galβ1-3GalNAcα1-Ser/Thr) (TF) is the consequence of other glycosyl transferases lack of integrity (30).

**Figure 3:**
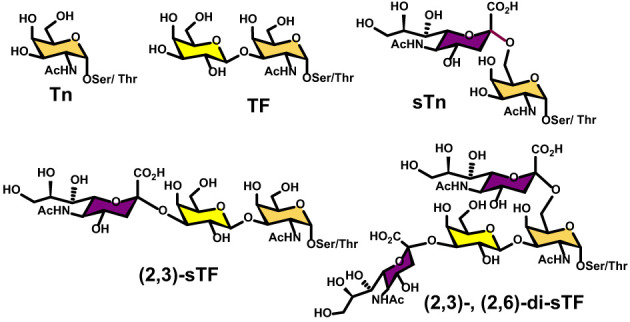
Structural details of the most common *O*-linked glycans present on both tumor cells and virus envelop glycoproteins.

Moreover, overexpression of α2,6- and α2,3-sialyl transferases result with the accumulation of the sialylated forms of the above two antigens resulting in 5 predominantly expressed TACAs structures illustrated in **Figure 3**: Tn, TF, sTn, (2,3)-sTF, and (2,3)-, (2,6)-di-sTF. These TACAs are usually present on 90% of carcinomas (31) and have been proposed as key glycoepitopes in various forms of cancer (32–35).

## 3 Glycan structures on enveloped viruses

Comprehensive identification, structural characterization, location, and impact of glycosylation on virus biology triggered new strategies to treat viral infections and has an increasing contribution on widespread vaccine design (36, 37). Documentation of consensus glycoepitopes in native viral glycoproteins and tumor cells is particularly useful in the conception of shared vaccination approaches (38).

**Figure 4:**
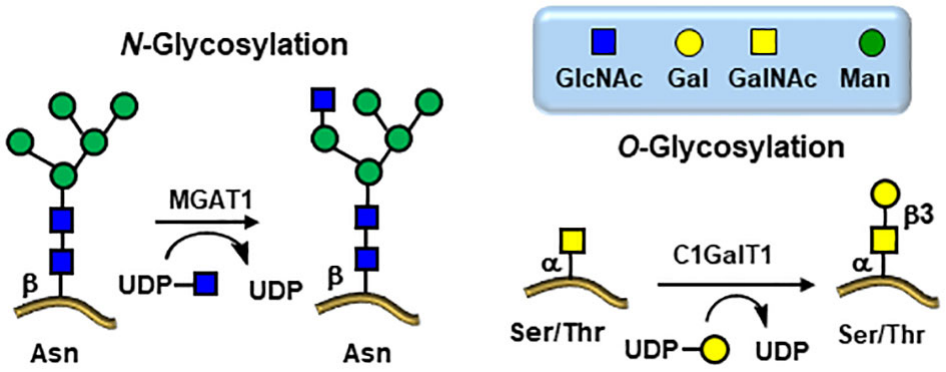
Viral glycosylation pathways hijacked from the infected host cells. Knock down MGAT1 and C1GalT1 glycosyltransferases inhibits viral entry.

Viral protein glycosylation is a pervasive post-translational modification that is responsible for virus protective shielding, host cell targeting, adhesion, and spreading (37). As obligate parasites such as some plants, fungi, and bacteria, viruses also exploit host-cell metabolic machinery to glycosylate their own proteins during replication (**Figure 4**) (36–39). Viral envelope glycoproteins from a variety of human pathogens, including influenza virus (40, 41), HIV-1 (42–44), Lassa virus (45), coronavirus (46–48), Zika virus (49), Dengue virus (50, 51), Ebola virus (52, 53), human respiratory syncytial virus (hRSV) (54), and more recently SARS-CoV-2 (55–58), have progressed to have been shown to be broadly glycosylated by both *N*- and *O*-linked glycoepitopes. These host cell-derived glycoforms facilitate diverse structural and functional roles during the viral life-cycle mostly related to immune evasion since these extensive glycosylation sites usually mask peptide sequences that would be otherwise useful targets for vaccine design (38, 39, 59). Importantly, whether the envelope glycoproteins of most of these viruses are expressed in human cells, their expression into other vectors lead to identical glycoform patterns (60).

**Figure 5:**
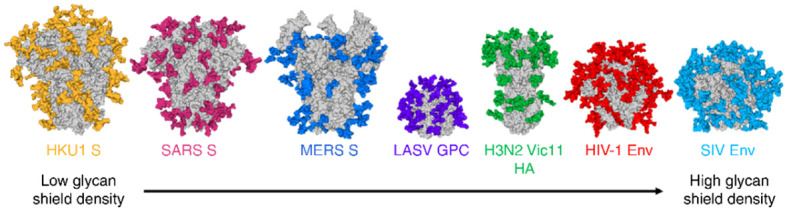
Typical high shielding effects of *N*-linked glycoepitopes (colored) of common viral envelope glycoproteins (the illustration shows mainly the *N*-glycosylation sites modeled on the Man_5_GlcNAc_2_ motif anchoring glycans). Reproduced with permission from reference (39).

Comprehensive structural investigations of the glycoepitopes of viral envelope glycoproteins have been investigated for a long time, particularly for *N*-linked glycans whose structural analysis were simpler (36–39). However, given the inherent technical difficulties encountered for the structural analysis of *O*-linked glycans, detailed studies of the latter have lagged behind (61, 62). Mass spectrometry has played and continues to play a vibrant role in chemical characterizations of both *N*- and *O*-linked glycans amongst glycomic and glycoproteomic analytical methodologies (62). As seen in **Figure 5**, *N*-glycans of enveloped viruses are so abundant that they clearly masked key amino acid epitopes (39), thus preventing the efficacy of several anti-peptide antibody approaches generated through common vaccinations of glycoproteins of enveloped viruses: human coronavirus: HKU1 S, SARS S, MERS S; Lassa virus glycoproteins (LASV GPC); influenza virus (H3N2); human immunodeficiency viruses (HIV-1; simian immunodeficiency viruses (SIV). Analogously to TACAs expressed on cancer cells, viral protein glycosylation patterns follow the same trends with typical fucosylation, sialylation, branching, and polylactosaminylation (22, 63, 64) in addition to their simplified *O*-linked glycan patterns.

*Glycan-Binding Proteins (Lectins) in Antiviral Therapies: The Envelope Glycoproteins of Influenza Virus.*

Lectins, *i. e*. carbohydrate-binding proteins of non-immune origin, play several key roles in biological processes, including innate immunity (65–67). In this regard, lectins act as pattern recognition receptors (PRRs) that can bind pathogen-associated molecular patterns (PAMPs). Consequently, like antibodies of the adaptative immune system, lectins greatly contribute to the protective immune responses. The major classes of lectins in innate immunity are represented by C-type lectin receptors (ex. Dectin-1, Langerin, Mincle, Ficolins, Dendritic Cell-Specific ICAM-3 Grabbing Integrin (DC-SIGN, L-SIGN, Mannose-Binding Protein (MBP), Galectins, and Siglecs (65–67). Alternatively, several leguminous lectins are also capable of binding glycans on both cancer cells and viruses (68).

The envelope glycoproteins of influenza viruses are mainly represented by their hemagglutinins (HA) and neuraminidases (NA) (69). Their *N*-glycosylation by the host machinery implies, like all other viruses, the attachment of oligosaccharides to the side-chain amide Asn at the Asn-X-Ser\Thr sequon, where X can be any amino acid except proline (70). The resulting glycan sequences involves high-mannose oligosaccharides, galactose and/or *N*-acetyl galactosamine/fucose (complex glycans) or a combination there of (hybrid glycans) (**Figures 1**, **5**). Consequently, typical lectins implicated in the innate immunity can bind, trigger viral neutralization, and prime the immune system. Unlike most antibodies, these lectins can bind to a wide range of influenza strains, implying that they would be attractive candidates for antiviral (“*immuno*’’) therapies (65, 67, 68, 71).

As mentioned above, most enveloped viral glycoproteins express the typical glycan structures detected on cancer cells. As such, they are inherently recognized by the endogenous lectins of the innate immune system with mannosides, galactosides, and sialic acid-ending glycans being the major players. Several such viral glycoproteins can also bind to leguminous lectins (**Table 1**). Thus, in spite of historically inadequate analytical mass spectrometry tools, leguminous lectins, together with C-type lectins of the innate immunity system have played central roles in the structural elucidation of glycans on viruses. The high mannose oligosaccharide (Man_9_GlcNAc_2_) of HIV-1 gp120 glycoprotein is highly representative of noteworthy efforts directed at generating glycoconjugate anti-viral vaccines (79).

**Table 1:** Human and plant lectins binding to glycoepitopes of viral envelop proteins.

Virus	Lectins	Sugar recognition	References
RSV	DC- & L-SIGN Lentil lectin *Helix pomatia* *Vicia villosa* B4 *Arachis hypogaea* *Lens culinaris* *Glycine Max (Soybean agglutinin) (SBA*	Mannose, LeX Mannose αGalNAc (Tn) αGalNAc (Tn) Gal-β(1-3)-GalNAcα (TF) Mannose αGalNAc (Tn)	(72) (72) (54, 73) (54, 73) (54, 73) (73) (74)
HIV, HCV	DC- & L-SIGN Griffithsin; Banana lectin Jacalin	Mannose Mannose Gal-β(1-3)-GalNAcα (TF)	(68) (75) (68)
HMPV	*Arachis hypogaea*	Gal-β(1-3)-GalNAcα (TF)	(76)
SARS-CoV-2	DC-SIGN Banana Lectin MGL Galectin-7 Galectin-3 Galectin-8 Siglec-8 Siglec-10	Mannose, LeX Mannose GalNAc LacNAc LacNAc 3’SLN 6’Sulfo-SLeX SLN	(77) (55–58) (77) (77) (77) (77) (77) (77)
Nipah virus	Galectin-1	Gal, GalNAc	(78)
Influenza A	Galectin-9, Galectin-1 Jacalin	Gal, GalNAc Gal-β(1-3)-GalNAcα (TF)	(78) (68)
Lassa virus Zika virus Ebola Dengue Coronavirus	DC- & L-SIGN, Ficolin, MBL, MGL, Banana lectin	Mannose, Gal	(45) (49) (52, 53) (50, 51) (46–48)

## 4 Detailed *O*-Linked glycans of the spike glycoproteins of SARS-CoV-2

**Figure 6:**
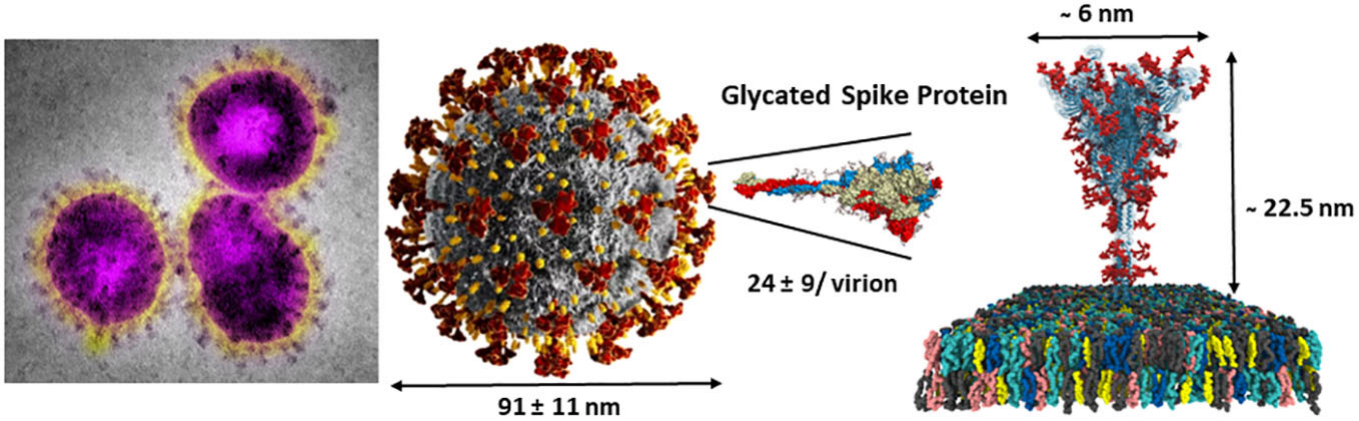
SARS-CoV-2 virus nanoparticles are coated by a large number of trimeric spike glycoproteins shielded by several *N*-linked glycans (red) and a few *O*-linked glycoepitopes (not shown) (see **Figure 7** for details). Right adapted from: https://charmm-gui.org/?doc=archive&lib=covid19.

**Figure 7:**
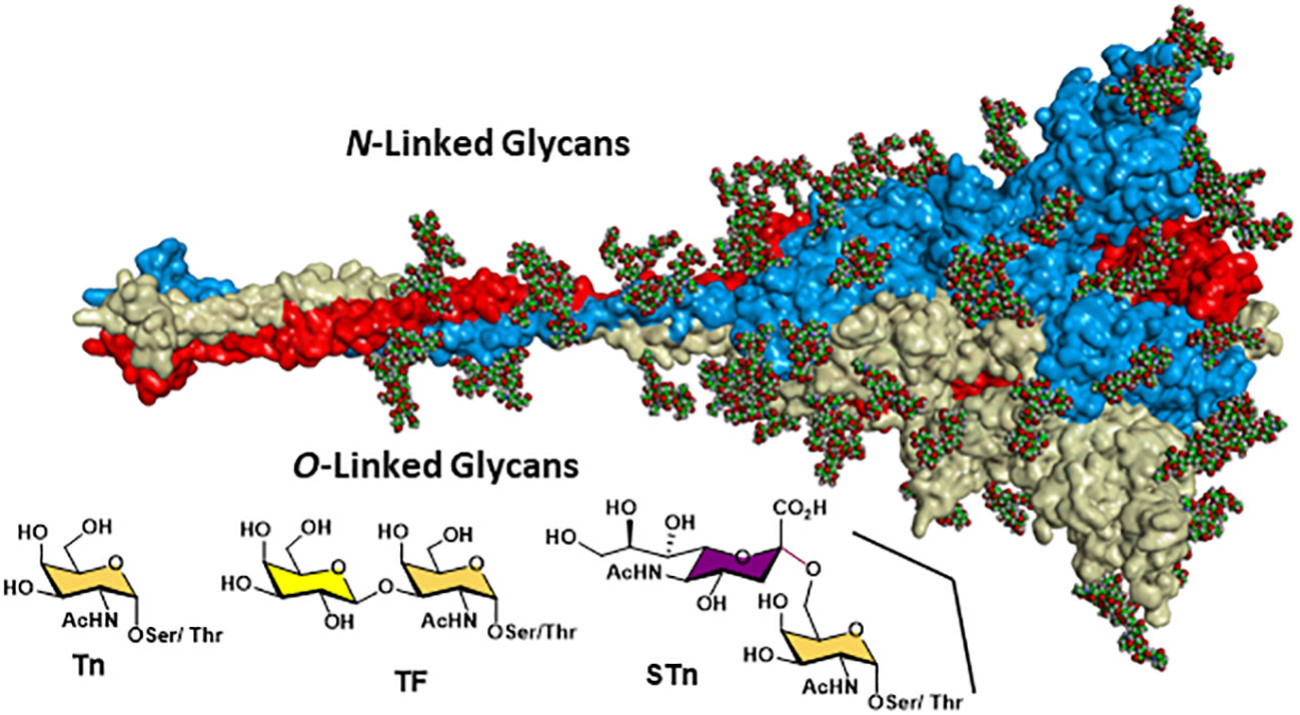
SARS-CoV-2 spike protein showing the dense shielding effect of the *N*-linked glycans (as CPK balls) and the region where the *O*-linked TACAs have been clearly identified using high-resolution LC-MS/MS ^320^VQPT*ES*IVR^328^, adapted from https://charmm-gui.org/?doc=archive&lib=covid19.

The scarcity of *O*-linked glycans on viral glycoproteins coupled with early difficulties in detailed structural analytical methodologies have impeded the progress of their detailed compositional and positional analyses (61, 62). However, the global pandemic of the SARS-CoV-2 in 2019 (80) has triggered tremendous efforts toward the discoveries of a wide range of new therapeutics strategies leading to investigations toward several potential targets, including glycan structures, and particularly in the field of vaccines (81, 82). The virion particles of SARS-CoV-2 are 91 ± 11 nm wide and covered with approximately 24 highly glycosylated fusion S-proteins which contain the binding site for its host cell receptor (RBD) – the angiotensin-converting enzyme 2 (ACE2) (**Figures 6**, **7**) (83). Further evidences for the key roles of proteins glycosylation on viruses were illustrated by the knocking out of two key enzymes responsible for the build-up of both *N*- and *O*-linked glycan biosynthesis (84). Yang et al. showed that the SARS-CoV-2 viral entry mechanism was inhibited by blocking α-1,3-mannosyl-glycoprotein 2-β-*N*-acetylglucosaminyl transferase (MGAT1) and the core 1 synthase, glycoprotein-*N*-acetylgalactosamine 3-β-galactosyltransferase 1 (C1GALT1) enzymes (**Figure 4**) using CRSP-Cas9 knockout cells (84).

Initially, it has been strongly speculated that sialic acid-ending residues acted as auxiliary co-receptor (85, 86), thus opening the door to sialic acid-based anti-adhesion inhibitors (87). More recently, sialylated glycolipids (gangliosides) have now been more accurately identified as the key natural host co-receptors (88). The heavily *N*-glycosylated spike protein of SARS-CoV-2 masked potentially immunogenic peptide sequences by forming dense shielding (see **Figures 5**, **6**).

Detailed site-specific glycan profiling of the betacoronavirus SARS-CoV-2 spike (S) glycoprotein has unraveled 17 *N*-glycan chains per monomer out of the 22 potential sites (55–58) thus covering 42% of the surface potentially accessible toward antibodies, much less than the surface covered by the glycans on HIV-1. The complete *N*-glycoform mapping has been thoroughly investigated using high-resolution LC-MS/MS (58). The same investigation (58) unambiguously revealed that the *O*-glycoforms were clearly identified at sites Thr323 and Ser 325, near the hinge region of the receptor-binding domain (RBD) domain of the S1 protein. Remarkably, the usual *O*-linked TACAs identified on tumor cells were also identified (Tn, TF, sTn, (2,3)-sTF, and (2,3)-, (2,6)-di-sTF) (**Figures 3**, **7**), together with some more elaborated chains. The presence of additional *O*-glycans at Ser673, Thr678, and Ser686 has also been postulated based on glycoinformatics (89). Deeper insights into the chemical details of the SARS-CoV-2 architectures with respect to the shielding of the glycans illustrate the effects of the carbohydrate glycoforms and the relative positioning of the TACAs of interest (90–94).

## 5 Joint strategies in the design of both TACAs and viruses therapeutic agents

As indicated, TACAs have long been identified on viral glycoproteins (36–39). Therefore, using viral carbohydrates as targets for neutralizing antibodies is offering several advantages for group-specific vaccine development since this approach could alleviate viral mutations commonly and rapidly occurring as it has been witnessed with the SARS-CoV-2 pandemic. One of the early reports of the presence of TACAs (sTn) on enveloped viruses has been reported on the gp120 of HIV isolates and anti-sTn antibodies were shown to neutralize infection of lymphocytes (95). Later, the same group also demonstrated that the related anti-Tn monoclonal antibodies (Mab) IgG2a (1E3) and an IgM (TKH6) inhibited both HIV-1 and HIV-2 infection *in vitro* (96). The antibodies showed a dose-dependent inhibition of viral infections together with syncytium formation in cultures inoculated with free virus. In addition, the infection was not prevented when synthetic α-GalNAc-Ser hapten was pre-incubated with the Mab 1E3 used to block the interaction. These findings are very relevant to our postulate since the presence of other *O*-linked glycans have been identified on other retroviral envelope glycoproteins (97).

**Figure 8:**
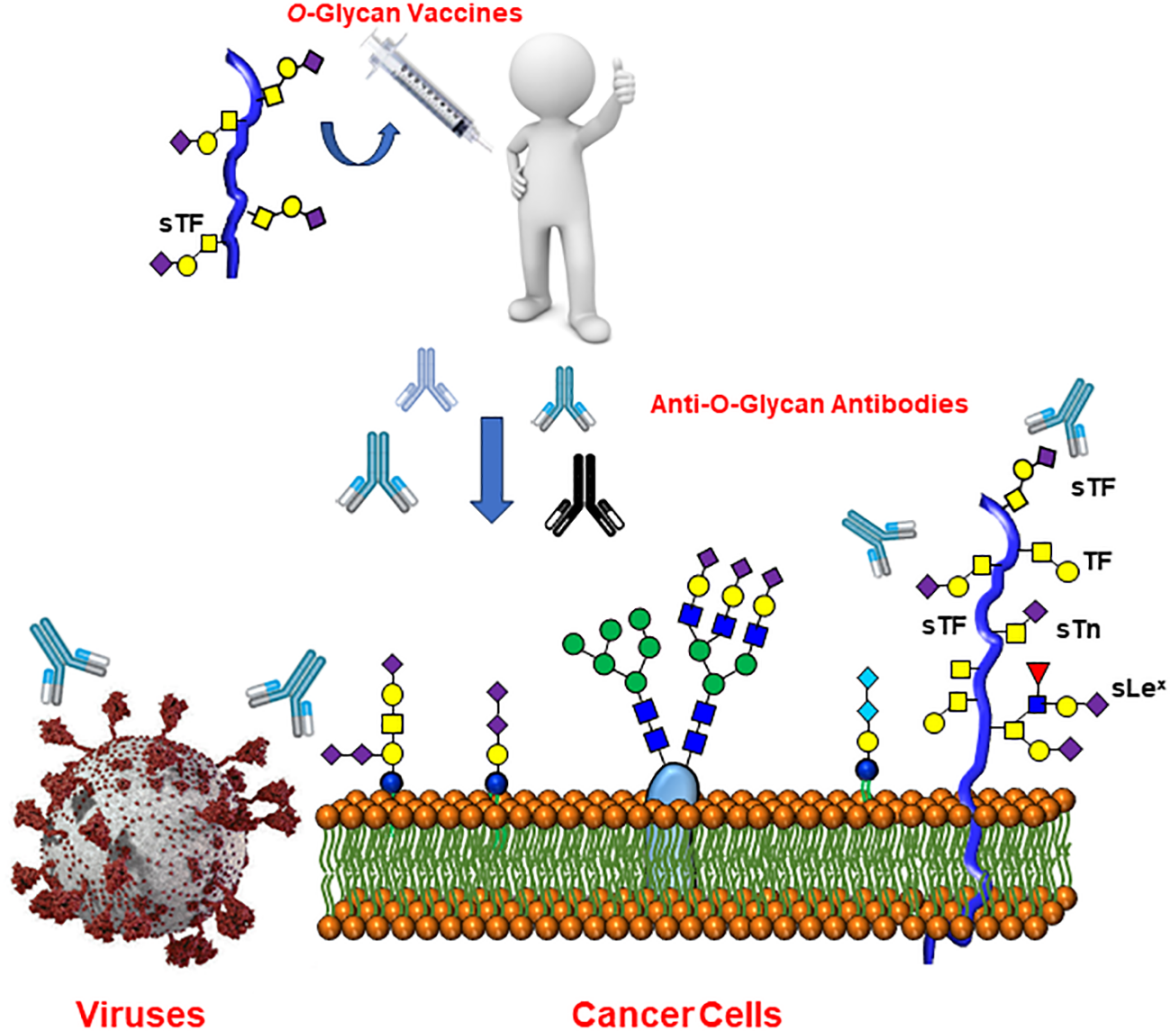
Raising anti-Tumor-Associated Carbohydrate Antigen (TACAs) antibodies may offer wide protection against both cancer and several forms of viral infections.

Importantly, it is well documented that human serum contains substantial amounts of a wide range of anti-carbohydrate antibodies. These have been identified using modern glycan microarrays (98–100) and has been postulated to originate from prior exposures of humans to various pathogenic agents, including viruses (101) and their relative contents and specificities depends on age and ethnicity (102). Interestingly, anti-TACAs antibodies were identified in the sera of healthy individuals (103) and it has been hypothesized that such antibodies might confer protection against COVID-19, as shown with the anti-Tn glycoepitope mentioned above (104). The study showed that lower levels of anti-Tn antibodies were specifically low in COVID-19 patients in comparison to those of the healthy group. These findings are not without precedent since the αGal glycoantigen constitute one of most extensively studied example of a carbohydrate epitope that can lead to the elimination of viruses through natural antibodies (105, 106). This glycoepitope is expressed by many cell types in most mammalian species, but is lacking in humans due to the lack of the cognate galactosyl transferase. Overall, natural anti-Tn antibodies could benefit from a natural immunity conferred by these antibodies against COVID-19 (and likely other viral infections) and corresponding vaccines would offer an attractive prophylactic perspective (**Figure 8**).

There are several other potential sources of carbohydrate-binding proteins (lectins) that could also be used against viral infections. Naturally occurring lectins from various organisms have been shown to inhibit viral entry mechanisms. These lectins are present in bacteria, plants, and marine algae (68). Lectins can block replications of viruses through binding with viral envelope glycoproteins. The molecular interactions rely on monosaccharides as well as complex branched glycans with a particular activity against high-mannose oligosaccharides as found on viral *N*-linked glycoproteins (**Figure 4**). However, the challenge in using heterologous lectins in clinical settings is limited by the complications of administrating foreign proteins into human system. Indeed, not only can they be immunogenic on their own but several lectins have displayed toxicity and mitogenic activities (107). Fortunately, a recent investigation demonstrated that a single amino acid substitution in a banana lectin (BanLec), replacing histidine 84 with a threonine, had significantly reduced its mitogenicity while preserving its broad-spectrum antiviral potency in HIV isolates (75). Interestingly, BanLec is known to be a mannose-specific lectin. In addition, studies have shown that BanLec can inhibit HIV-1 reverse transcriptase activity, suppress influenza viral fusion, and provide protective activity against herpes simplex virus (HSV) type 1 (108). Additionally, it has been observed to suppress cancer cell proliferation (109). Furthermore, and in line with the above relationships between TACAs on cancer cells and the ability of leguminous lectins to recognize tumors, a recent report described the use of BanLec CAR-T cells to target pancreatic tumors (110).

Most significantly, human innate immunity, conferred by the family of mannose-binding lectins (MBLs) such as the ficolins and the membrane-bound CD209 (DC-SIGN) similarly constitute protective activities against viral infections (67). The complement cascade of activation is also playing a key role in the initial defense mechanism against viral infections (111). These mannose-binding lectins are usually abundant in healthy human serum (1340 ng/mL) (112). Humans deficient in their serum concentrations of MBLs were shown to be more susceptible to SARS-CoV-2 infections (113).

Interestingly, non-peptidic carbohydrate binding agents (CBAs) such as pradimicin A and benanomicin A family of antibiotics produced in the actinomycetes strain *Actinomadura hibisca* and *Actinomodura spadix*, respectively, known to bind mannose-containing oligosaccharides, have also been shown to be potent inhibitors of viral infections (114, 115). They can also inhibit syncytia formation as well as DC-SIGN-mediated transmission of HIV to lymphocytes.

## 6 Conclusion

There are an increasing number of evidences clearly pointing toward common glycoepitopes present on both cancer cells and viral envelope glycoproteins. Several studies have established beneficial effects of monoclonal anti-carbohydrate antibodies against tumor-associated carbohydrate antigens (TACAs) as well as specific carbohydrate-binding lectins to protect entry and viral replications from a wide-range of envelope viruses. This report illustrated numerous examples wherein leguminous lectins were used to identify the presence of *O*-linked glycans (TACAs) on a wide range of viruses. However, further evidences are required to demonstrate that antibodies equivalent to lectin-recognition domains would be as efficacious as immunoprophylactics. Therefore, this report highlights the necessity to deepen our understanding on the vaccinal applications of existing MAbs against TACAs that can be potentially and equally useful as anti-viral vaccine prophylactics (**Figure 8**).

## Author contributions

RR: Writing – review & editing.
